# Constraining shifts in North Atlantic plate motions during the Palaeocene by U-Pb dating of Svalbard tephra layers

**DOI:** 10.1038/s41598-017-06170-7

**Published:** 2017-07-28

**Authors:** Morgan T. Jones, Lars E. Augland, Grace E. Shephard, Seth D. Burgess, Gauti T. Eliassen, Malte M. Jochmann, Bjarki Friis, Dougal A. Jerram, Sverre Planke, Henrik H. Svensen

**Affiliations:** 1 Centre for Earth Evolution and Dynamics (CEED), University of Oslo, PO Box 1028 Blindern, 0315 Oslo, Norway; 2 Massachusetts Institute of Technology, 77 Massachusetts Avenue, Cambridge, MA 02139-4307 USA; 3 Store Norske Spitsbergen Grubekompani, Postboks 613, 9171 Longyearbyen, Svalbard Norway; 4 The University Centre in Svalbard (UNIS), P.O.Box 156, 9171 Longyearbyen, Norway; 5 DougalEarth Ltd., 31 Whitefields Crescent, Solihull, B91 3NU UK; 6 Volcanic Basin Petroleum Research (VBPR AS), Forskningsparken, Gaustadalléen 21, 0349 Oslo, Norway; 7 Present Address: California Volcano Observatory, Volcano Science Center, United States Geological Survey, 345 Middlefield Road, Mail Stop 910, Menlo Park, CA 94025 USA

## Abstract

Radioisotopic dating of volcanic minerals is a powerful method for establishing absolute time constraints in sedimentary basins, which improves our understanding of the chronostratigraphy and evolution of basin processes. The relative plate motions of Greenland, North America, and Eurasia changed several times during the Palaeogene. However, the timing of a key part of this sequence, namely the initiation of compression between Greenland and Svalbard, is currently poorly constrained. The formation of the Central Basin in Spitsbergen is inherently linked to changes in regional plate motions, so an improved chronostratigraphy of the sedimentary sequence is warranted. Here we present U-Pb zircon dates from tephra layers close to the basal unconformity, which yield a weighted-mean ^206^Pb/^238^U age of 61.596 ± 0.028 Ma (2σ). We calculate that sustained sedimentation began at ~61.8 Ma in the eastern Central Basin based on a sediment accumulation rate of 71.6 ± 7.6 m/Myr. The timing of basin formation is broadly coeval with depositional changes at the Danian-Selandian boundary around the other margins of Greenland, including the North Sea, implying a common tectonic driving force. Furthermore, these stratigraphic tie points place age constraints on regional plate reorganization events, such as the onset of seafloor spreading in the Labrador Sea.

## Introduction

**Figure 1:**
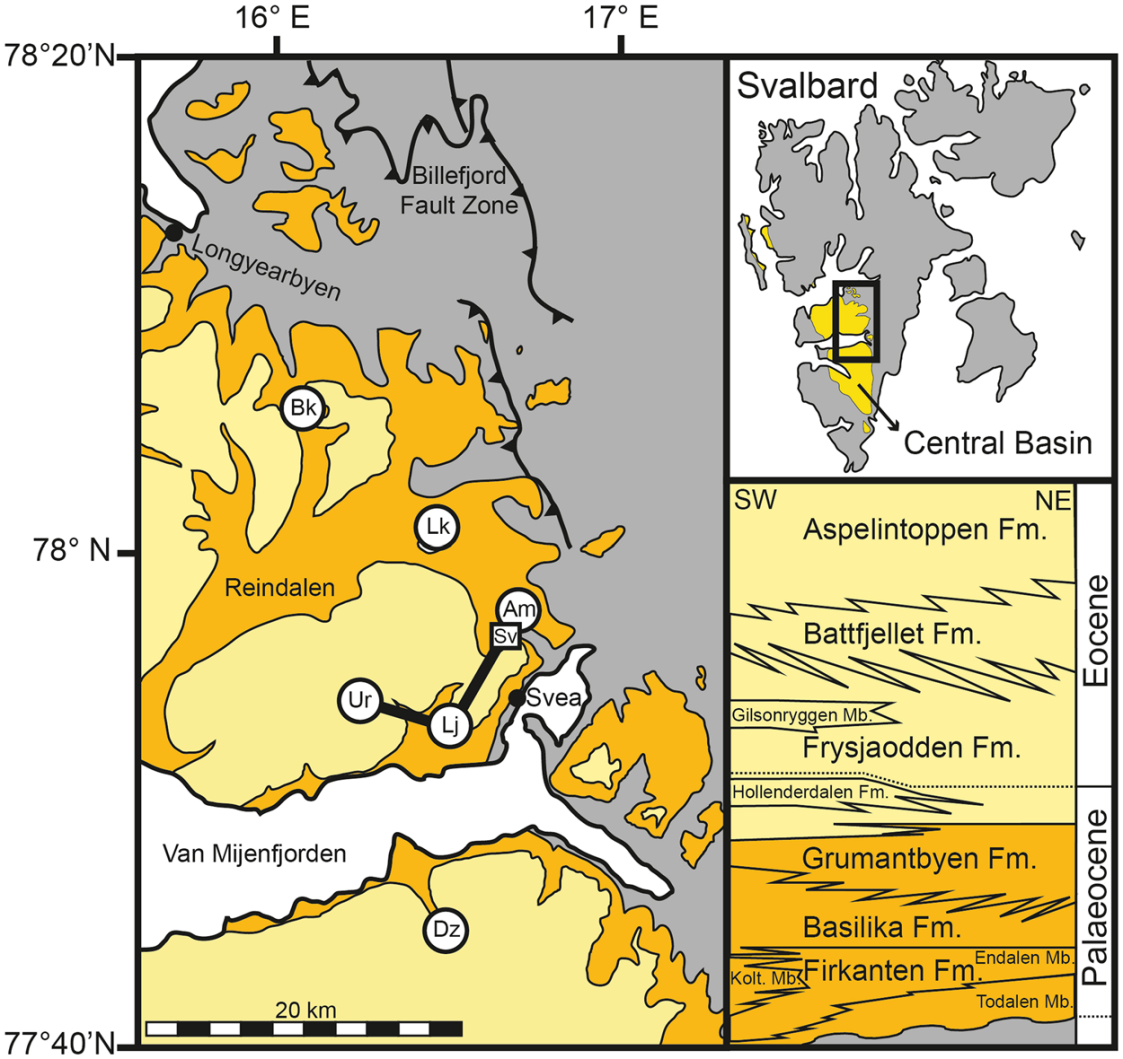
A map of the study area, the eastern Central Basin in Svalbard. The names and positions of the cores are labelled with abbreviations: Bolterskaret = Bk; Lunckefjellet = Lk; Amelnryggen = Am; Svea mine = Sv; Liljevalchfjellet = Lj; Urdkollen = Ur; Danzigdalen = Dz (see Fig. 3 for details). The lithostratigraphic inlay shows the main formations of the Van Mijenfjorden Group^5^. The three lowermost formations are shown in orange (Palaeocene), the four uppermost formations in light yellow (latest Palaeocene to Eocene), and pre-Cenozoic stratigraphy in grey. The lines between core locations ‘Ur’, ‘Lj’, and ‘Am’ represent the trace of the cross section (Fig. 3). The map and geological boundaries are manually redrawn using geological maps of Svalbard^55^ (© Norwegian Polar Institute; www.npolar.no).

The onset of compression between Greenland and Svalbard in the Palaeocene led to the eventual formation of the West Spitsbergen fold-and-thrust belt in the Eocene, with a rapidly subsiding foreland basin forming adjacent to the mountain range^1–3^ (Fig. 1). The Central Basin is of particular interest because the timing of the basin’s formation is inherently linked to the evolution of regional tectonics and the relative motions of North America, Greenland, and Eurasia, which changed significantly during the Palaeogene^4^. The basin infill is named the Van Mijenfjorden Group; a 2.3 km thick succession subdivided into seven formations and one subgroup^5^ that are predominantly sandstones and siliciclastic mudstones deposited in fluvial, deltaic, and marine shelf environments^6^ (Fig. 1). The West Spitsbergen fold-and-thrust belt is part of a larger complex across northern Greenland and Ellesmere Island that constitutes Eurekan deformation (~53–34 Ma)^3, 7^. Subsidence in the Central Basin began before the formation of the fold-and-thrust belt and with no clear hiatus in sediment deposition. Therefore, a detailed understanding of the basin stratigraphy can be used to refine the chronology of plate reconfigurations in the run up to the Eurekan deformation and the opening of the northeast Atlantic.

Constraining the temporal evolution of the Central Basin is complicated by the scarcity of age-specific fauna and flora in the sedimentary record^8^ and a paucity of radioisotopic dates. The basin’s enclosed nature during the Palaeocene and early Eocene led to low salinity and locally low oxygen conditions, resulting in water column stratification with high carbonate corrosivity^6^. Biostratigraphic comparisons with global datasets are hampered by the distinct Boreal biogeographic province in the Arctic due to limited deep water connections to other oceans^9^. Radioisotopic dating of tephra layers in the Palaeocene Central Basin strata is limited. A ^206^Pb/^238^U age of 55.785 ± 0.086 Ma was obtained from a tephra horizon within the early Eocene Frysjaodden Formation^10^, the fourth formation within the Van Mijenfjorden Group^5^ (Fig. 1). This layer provides the basis for anchoring local cyclostratigraphic age models and the timing and duration of the Palaeocene-Eocene Thermal Maximum (PETM)^10^. The two lowermost formations (Firkanten and Basilika; Fig. 1) contain numerous bentonite (altered tephra) layers that could potentially be used for radioisotopic dating. Previous investigations that attempted to obtain radioisotopic ages for the Basilika formation found only detrital/inherited zircon grains, despite analysing a total of 430 zircons from four bentonite horizons^11^. The lack of accurate depositional ages, coupled with the importance of this location in constraining the first sustained compression between Greenland and Svalbard, highlights the need for an improved geochronology of the Central Basin.

## Results

**Figure 2:**
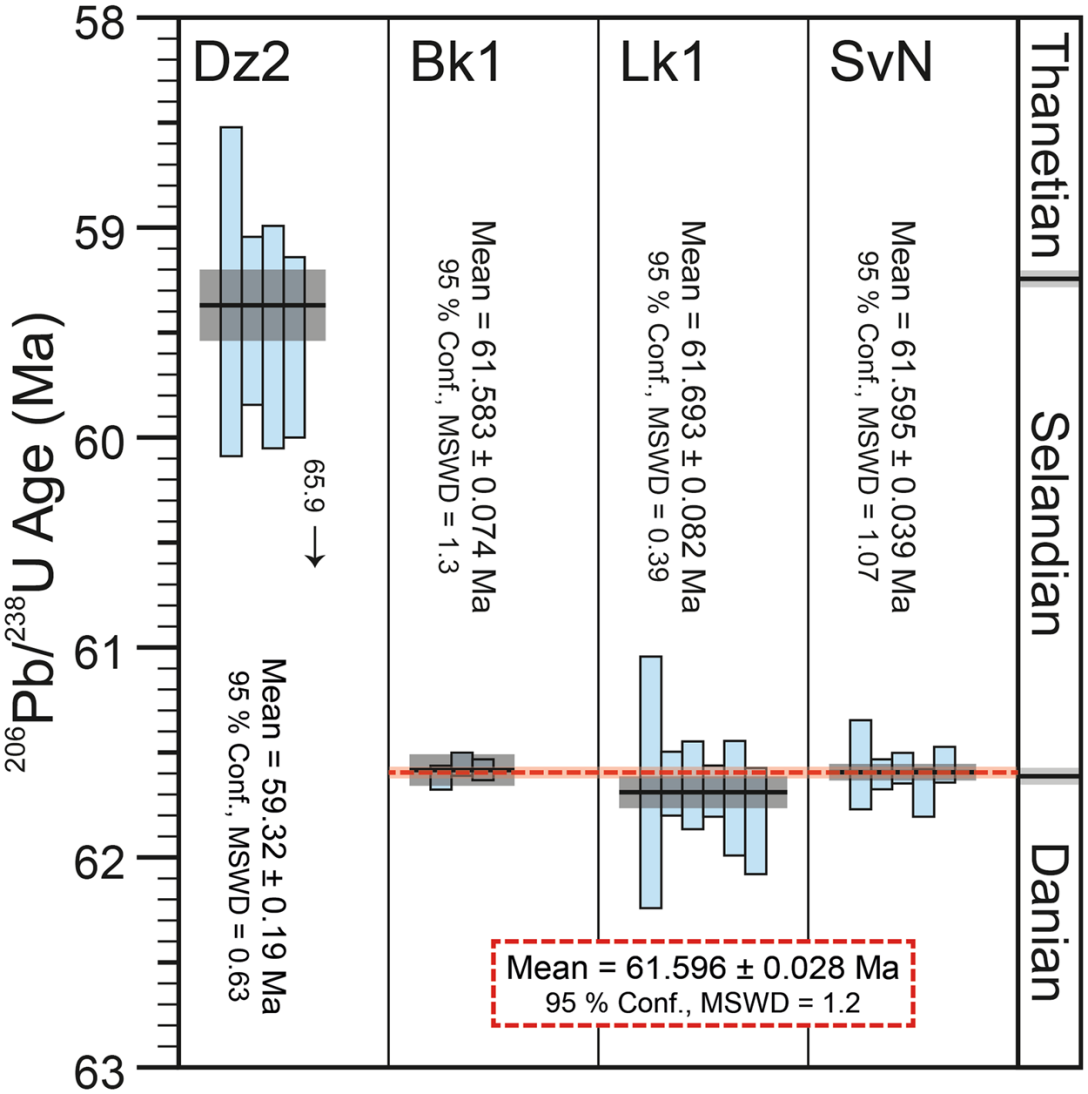
Single-grain and weighted mean ^206^Pb/^238^U dates for Central Basin bentonites. The height of the single grain rectangles (light blue) is proportional to the 2σ uncertainty of that measurement. The weighted mean of each sample set is shown as a black line, with translucent grey bands either side representing the 2σ uncertainty. The combined mean age for sample ‘Bk1’, ‘Lk1’, and ‘SvN’ (equivalent to sample ‘Am1’^13^) is shown as a red dashed line through samples with 2σ uncertainty shown as a translucent red band. Astronomically calibrated Palaeocene stage boundaries^25^ are shown on the right.

**Figure 3:**
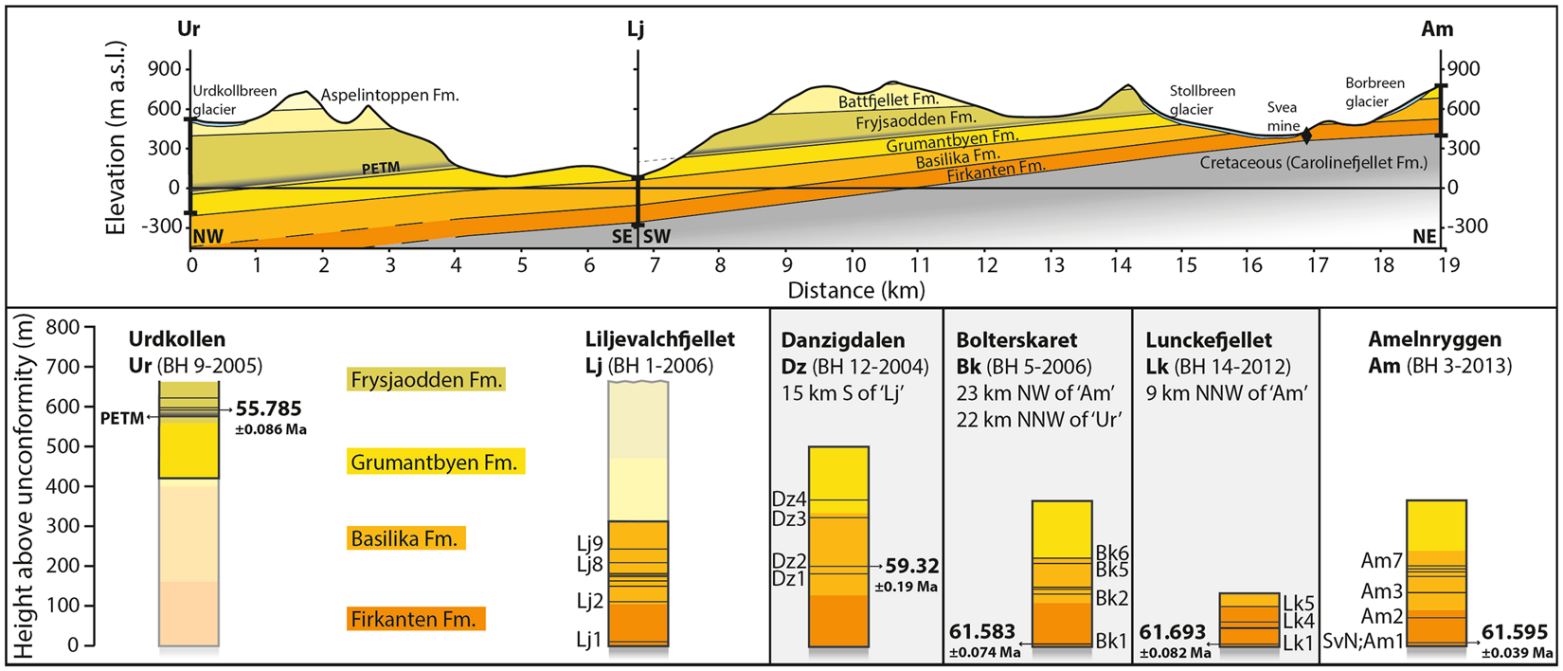
An idealised cross-section (2x vertical exaggeration) and core logs of the study area based on field outcrops, geological maps^55^, and borehole information. The positions of the cross section points are shown in Fig. 1. The locations and depths of cores ‘Ur’, ‘Lj’, and ‘Am’ are shown as thick black lines. The Svea mine entrance is denoted with a black diamond on the cross-section. The core logs below are normalised to the basal unconformity, with ash layers shown as black lines. Dated horizons are labelled. Translucent parts of cores ‘Ur’ and ‘Lj’ are inferred from field evidence.

Euhedral, core-free (optically inspected) zircon crystals interpreted to be of magmatic origin were isolated from four bentonite layers within the Firkanten and Basilika formations; three from separate borehole cores and one from an outcrop inside the Svea coal mine (Fig. 1). Single zircon crystals were dated by the U-Pb isotope dilution thermal ionization mass spectrometry technique (ID-TIMS; see Methods Section). Individual dates and weighted-mean ^206^Pb/^238^U ages are shown in Fig. 2, with data presented in Table 1. The core abbreviations are described in Figs 1 and 3. Three of the dated horizons (Bk1, Lk1, SvN) and samples Am1 and Lj1 are from a prominent ash layer located between 7.1 and 11.7 m above the regional Lower Cretaceous-Palaeocene low-angle (0.5–1°)^5^ unconformity (Fig. 3). Weighted-mean ^206^Pb/^238^U ages for the three samples are between 61.693 ± 0.082 Ma and 61.583 ± 0.074 Ma (2σ; including uncertainties in tracer calibrations but excluding decay constant errors). Outcrop evidence from within the Svea mine and in the field strongly suggests that these samples are from the same laterally continuous bentonite horizon that represented a large single eruption^12^. This is corroborated by matching geochemical signatures between samples despite extensive alteration^13^. The bentonite is not present in all cores in the basin, likely due to subsequent reworking that led to the erosion of tephra from palaeo-topographic highs^12^. However, close inspection of sedimentary structures within each bentonite layer shows no evidence of a hiatus in deposition, nor variations in chemistry from base to top^13^. This indicates that any reworking of material occurred soon after deposition and it is therefore extremely likely that these layers represent a single source. Assuming that the three samples (Bk1, Lk1, SvN) are equivalent, combining these analyses yields a weighted-mean ^206^Pb/^238^U age of 61.596 ± 0.028 Ma (Fig. 2). As with previous studies^11^, tephra horizons from the Basilika Formation were dominated by detrital grains. Only one sample (Dz2) was found to contain Palaeocene zircons, with a weighted mean ^206^Pb/^238^U age of 59.32 ± 0.19 Ma (Fig. 2). This layer is 203.8 m above the unconformity and 73.7 m above the Firkanten-Basilika boundary (a change from sandstone-dominated to shale-dominated deposition^5^). The lower precision on the age of this bentonite is partly due to the smaller grain size of zircons (<90 µm; zircons from the basal tephra are ~150–200 µm).

**Table 1:** (a) Model Th/U ratio calculated from radiogenic ^208^Pb/^206^Pb ratio and ^207^Pb/^235^U age.

Compositional Parameters				Radiogenic Isotope Ratios							Isotopic Ages					
An. no	$\frac{{\bf{Th}}}{{\bf{U}}}$	Pb_c_ (pg)	$\frac{{{\bf{Pb}}}^{{\boldsymbol{\ast }}}}{{\bf{Pbc}}}$	$\frac{{}^{{\bf{207}}}{\bf{P}}{\bf{b}}}{{}^{{\bf{206}}}{\bf{P}}{\bf{b}}}$	% err	$\frac{{}^{{\bf{207}}}{\bf{P}}{\bf{b}}}{{}^{{\bf{235}}}{\bf{U}}}$	% err	$\frac{{}^{{\bf{206}}}{\bf{P}}{\bf{b}}}{{}^{{\bf{238}}}{\bf{U}}}$	% err	corr. coef.	$\frac{{}^{{\bf{207}}}{\bf{P}}{\bf{b}}}{{}^{{\bf{206}}}{\bf{P}}{\bf{b}}}$	- ±	$\frac{{}^{{\bf{207}}}{\bf{P}}{\bf{b}}}{{}^{{\bf{235}}}{\bf{U}}}$	- ±	$\frac{{}^{{\bf{206}}}{\bf{P}}{\bf{b}}}{{}^{{\bf{238}}}{\bf{U}}}$	-±
	(a)	(b)	(b)	(c)	(d)	(c)	(d)	(c)	(d)		(e)	(d)	(e)	(d)	(e)	(d)
**Lk1 (BH 14-2012)**																
1	0.89	1.7	6.1	0.0482	2.8	0.0639	3.0	0.009608	0.98	0.374	110	67	62.6	1.9	61.64	0.60
2	0.69	0.6	17.2	0.04778	1.0	0.0633	1.1	0.009609	0.25	0.436	88	24	62.32	0.65	61.65	0.15
3	0.55	1.1	6.9	0.0483	2.5	0.0641	2.6	0.009637	0.41	0.304	113	59	63.1	1.6	61.83	0.25
4	1.10	0.7	6.2	0.0488	3.2	0.0648	3.3	0.009620	0.45	0.327	140	75	63.7	2.1	61.72	0.27
5	0.69	0.8	23.5	0.04735	0.6	0.0628	0.6	0.009615	0.20	0.469	67	13	61.82	0.38	61.69	0.12
**Dz2 (BH 12-2004)**																
1	n.m	0.7	0.3	0.0531	16.7	0.075	18	0.01028	1.06	0.806	332	379	74	12	65.93	0.69
2	0.72	1.5	0.6	0.051	24.0	0.065	25	0.00924	1.30	0.828	228	554	64	15	59.30	0.76
3	0.87	0.7	2.2	0.0432	7.61	0.0549	7.9	0.009225	0.47	0.707	−159	189	54.3	4.2	59.20	0.28
4	0.56	0.5	1.6	0.0465	10.5	0.059	11	0.009264	0.65	0.767	20	252	58.6	6.3	59.45	0.38
5	0.82	1.0	0.8	0.0436	18.5	0.056	19	0.009276	0.86	0.816	−135	457	55	10	59.52	0.51
**Bk1 (BH 5-2006)**																
1	0.73	1.4	17.3	0.04780	1.0	0.0632	1.0	0.0095913	0.085	0.235	88	23	62.21	0.59	61.617	0.055
2	0.71	1.0	19.0	0.04763	0.9	0.06289	0.9	0.0095814	0.083	0.240	80	21	61.93	0.55	61.555	0.053
3	0.76	0.8	25.3	0.04757	0.7	0.06284	0.7	0.0095859	0.072	0.217	77	16	61.88	0.41	61.581	0.048
**SvN (Svea mine; Am1)**																
1	0.73	1.0	3.6	0.0481	4.6	0.0635	4.7	0.009595	0.35	0.237	104	109	62.6	2.8	61.56	0.21
2	0.70	0.9	11.2	0.04788	1.5	0.0633	1.5	0.009602	0.12	0.242	92	35	62.30	0.92	61.605	0.072
3	0.80	0.8	11.8	0.04760	1.4	0.0629	1.5	0.009598	0.12	0.256	78	34	61.92	0.88	61.575	0.073
4	0.71	1.1	7.3	0.04798	2.3	0.0635	2.3	0.009616	0.18	0.235	97	54	62.5	1.4	61.69	0.11
5	0.70	0.7	9.7	0.04765	1.7	0.0629	1.8	0.009595	0.14	0.236	81	41	62.0	1.1	61.559	0.085

(b) Pb* and Pbc represent radiogenic and common Pb, respectively. n.m.: Not measured (c) Corrected for fractionation, spike, and common Pb; all common Pb was assumed to be procedural blank. (d) Errors are 2σ, propagated using published algorithms^51^. (e) Calculations are based on published decay constants^54^. ^206^Pb/^238^U and ^207^Pb/^206^Pb ages corrected for initial disequilibrium in ^230^Th/^238^U using Th/U [magma] = 3.

## Discussion

**Figure 4:**
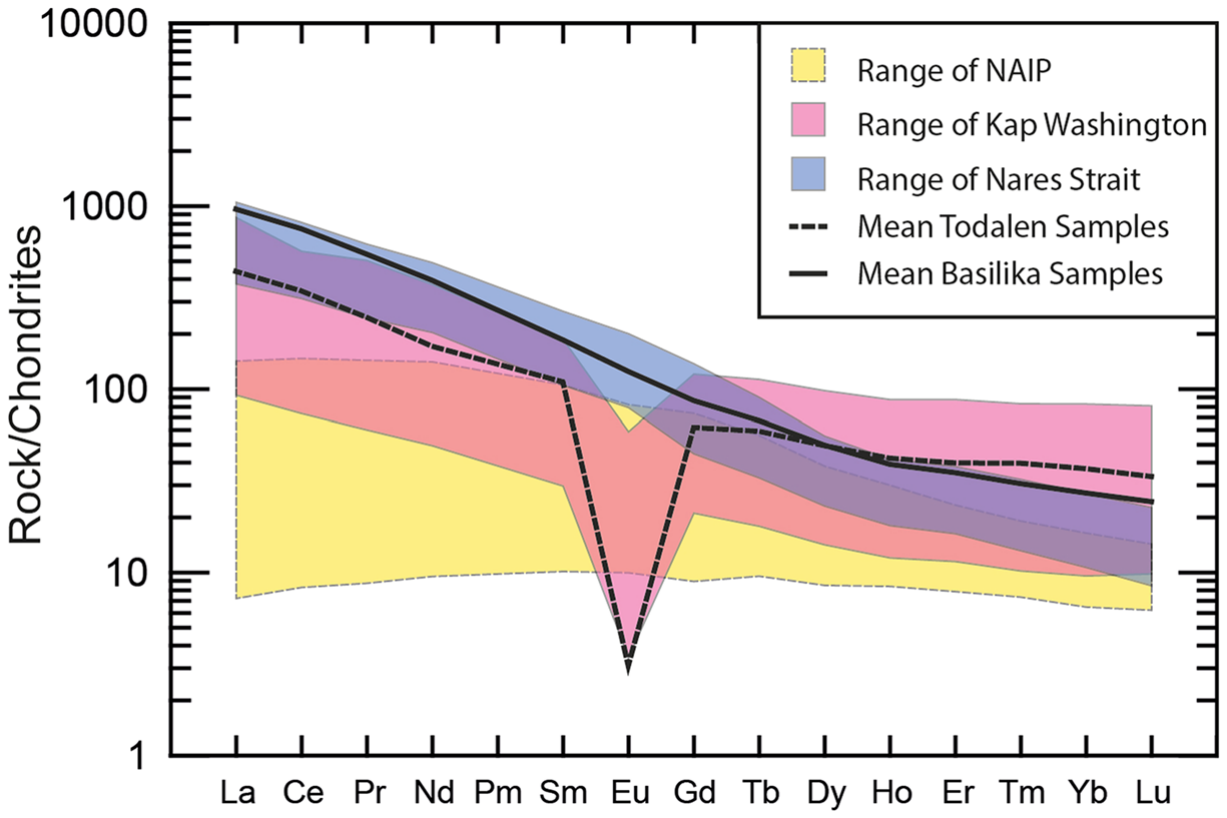
A summary of chondrite-normalised^56^ REE data, showing the mean values of the basal Todalen bentonite (dashed line) and later Firkanten and Basilika tephras (solid line)^13^. For comparison, the range of values from the Palaeocene exposures of the NAIP from East and West Greenland^19, 20^ are shown in yellow, the range in values from the Kap Washington Group in North Greenland^14^ in pink, and the range of volcaniclastic deposits in Ellesmere Island attributed to the Nares Strait^17^ in blue. All Central Basin samples show relative REE enrichment with respect to the entire range of measured NAIP rocks.

Previous investigations have established that the bentonites from the Firkanten and Basilika formations originated from at least two alkaline volcanic sources that are a likely product of continental rifting^11, 13^. Samples from the basal tephra layer are chemically distinct from all later ashes found in the cores, displaying overall REE enrichment with respect to chondrite, a moderate enrichment in LREE compared to HREE, and pronounced negative Ti, P, and Eu anomalies^13^. Full details of the geochemical fingerprinting of these tephra layers are presented in a previous study^13^. These chemical signatures closely match the ignimbrites and lavas of the Kap Washington Group in North Greenland^14^, indicating that this series is the most likely source (Fig. 4). The U-Pb ages determined herein are within the range of U-Pb ages derived from Kap Washington exposures (71–61 Ma)^15^, suggesting that this horizon likely represents one of the later explosive eruptions from this volcanic group. The close proximity of the tephra layer to the basal unconformity (7.1–11.7 m) corroborates the hypothesis that the cessation of Kap Washington volcanism was broadly contemporaneous with the formation of the Central Basin^16^.

The younger bentonites in the Firkanten and Basilika formations also display strong REE enrichment compared to chondrite, but display a strong enrichment of LREE compared to HREE and have little to no Eu anomalies (Fig. 4)^13^. These distinctive rare earth chemical signatures are comparable to volcaniclastic deposits in Ellesmere Island of suspected Nares Strait origin^17^, which suggests that the most likely source volcanoes of the younger suite of Svalbard tephras originated from the Nares Strait^17, 18^. None of the tephra layers studied here match the compositions of North Atlantic Igneous Province (NAIP) deposits from either West or East Greenland during the first phase of NAIP activity in the Palaeocene (Fig. 4)^19, 20^, negating this activity as a possible source. However, other unidentified sources proximal to the Arctic cannot be discounted for some layers^11^. Volcaniclastic deposits of suspected Nares Strait origin have been dated via ^40^Ar/^39^Ar to 61–58 Ma^17^, in agreement with the U-Pb age obtained here. There is no apparent temporal overlap of explosive activity between the two alkaline suites preserved in the Central Basin, with between 40 to 125 m of sediment between the lowermost Firkanten ash and the first appearance of the second group of bentonites in the studied cores^13^ (Fig. 3).

Radioisotopic dates from the Firkanten and Basilika formations, coupled with an U-Pb age from the Frysjaodden Formation^10^, allows us to estimate sediment accumulation rates. A cross-section across the region shows that the thickness of each formation increases westwards and southwards (Fig. 3). The prominent lowermost bentonite found in the eastern cores is absent from the western cores studied^13^, negating the possibility of correlating across all parts of the basin. Cross-section correlations using the new and published^10^ ages give an integrated sediment accumulation rate of 71.6 ± 7.6 m/Myr for the Firkanten and Basilika formations at Liljevalchfjellet after compaction (Fig. 3). The estimated mean sediment accumulation rate between the dated Basilika layer and the previously dated Frysjaodden bentonite^10^ is 90.9 ± 7.7 m/Myr at Liljevalchfjellet after compaction (Fig. 3). These errors include radioisotopic analytical uncertainties, coupled with errors associated with comparing different formation thicknesses between cores.

Extrapolated sediment accumulation rates allow for an estimation of first deposition in the eastern Central Basin. The first Palaeocene sediment deposition on the Cretaceous peneplain was the Grønfjorden bed, a conglomerate that is thought to represent braided river deposits in low-relief ridge and valley systems^21^. The Grønfjorden bed thickens to the southwest, which combined with the dominant source of clastic infill from the east^11, 22^, and the initiation of sedimentation in new areas in step-wise fashion during major transgressions^23^, strongly suggest the basal unconformity is not isochronal. Field, mine, and core examination indicates a shallow cross cutting relationship between the lowermost bentonite and the prominent Svea coal seam, indicating that the coal deposition was slightly diachronous with a northward migration of the swamps and peat mires through time^12, 13^. The bentonite is located 3.5 to 6.6 m above the coal seam in the area considered but is found below the coal seam in cores further north^12^. If it is assumed that the calculated sedimentation rate for the Firkanten formation is the same below the lowermost bentonite, then extrapolation allows us to infer that the first sediment deposition at Liljevalchfjellet is calculated to be 61.76 ± 0.09 Ma (using the same error propagation described above). However, given the difference in lithologies below (largely conglomerate and coal) and above (delta plain clastic sediments) the lowermost bentonite, the compaction of peat mires to form coal beds, and lithological evidence that deposition began southwest of the area studied, the timing of first deposition in the Central Basin began earlier than this estimate. What can be concluded with some certainty is that the lowermost bentonite is broadly coeval with the beginning of sustained and increasingly rapid subsidence in response to compression between Greenland and Svalbard.

**Figure 5:**
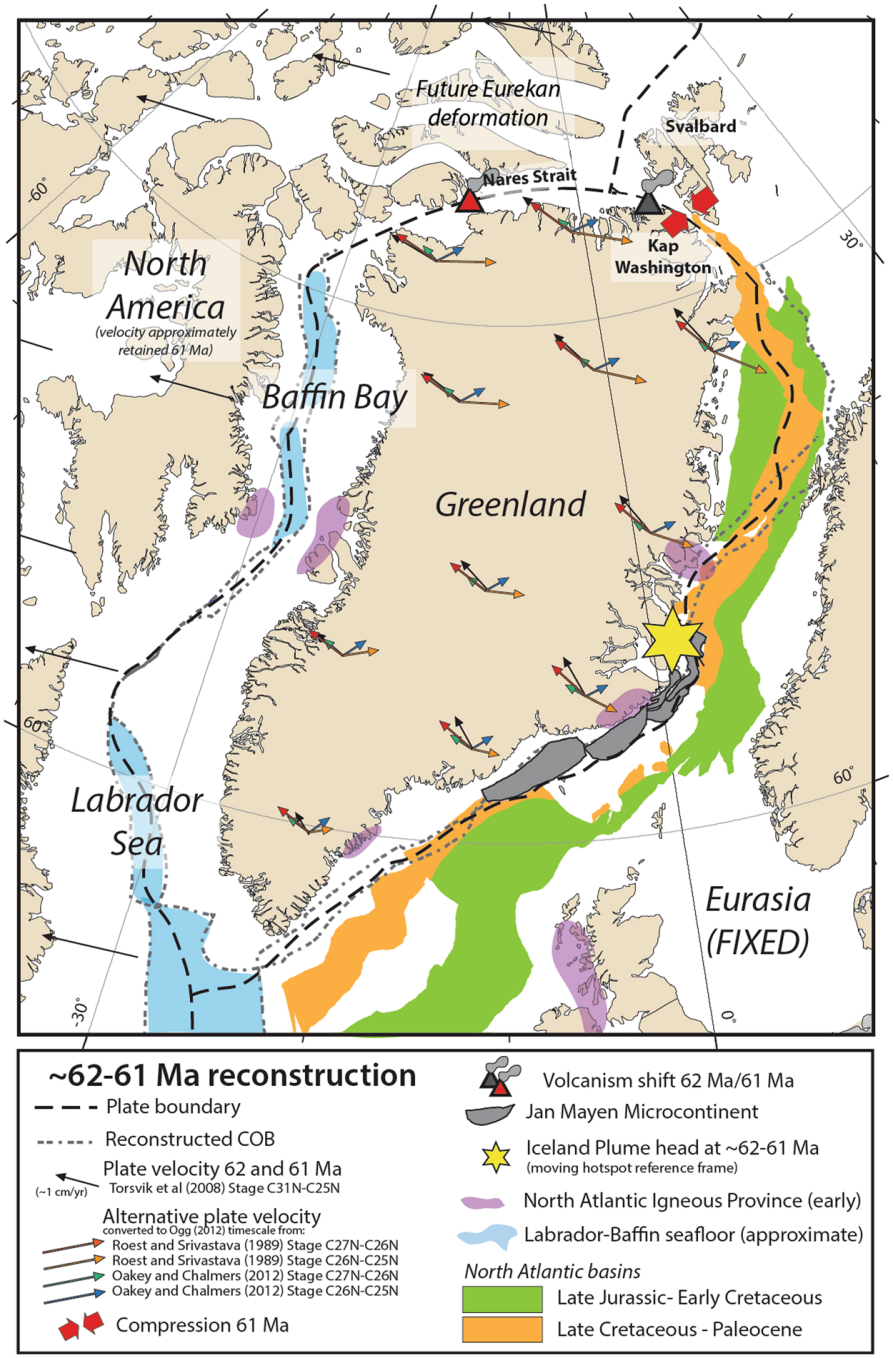
A regional plate reconstruction in a fixed Eurasia reference frame at 62–61 Ma. The proposed plate reorganisation, showing a transition from strike-slip to compression in Svalbard, and a shift in volcanism from Kap Washington to the Nares Strait, are shown as grey features before (62 Ma) and red features after (61 Ma). The black arrows represent plate velocities at 62 Ma (and 61 Ma, unchanged), based on the published stage rotation from C31-C25 from ref. [40]. Red and orange arrows correspond to single million year velocities derived from published stage rotations of C27-C26 and C26-25, respectively, for ref. 31. The green and blue arrows, as above, but derived from ref. 30. Models converted to the geomagnetic timescale of ref. 37 (young end of normal chron). Early NAIP activity^34^ is shown in purple. The duration of the stage pole for Greenland at 61 Ma, related to Palaeocene compressional features^33^, and magnitude of velocities are uncertain. The figure was created with open source plate tectonic software GPlates^57, 58^ based on published modifications of the reconstruction model^59^ and plotted with Generic Mapping Tools^60^.

The radioisotopic dating of bentonites from the Central Basin allow for an improved understanding of the first Palaeocene compressional motion between Greenland and Svalbard in the ‘pre-Eurekan deformation’ stage^3^ (Fig. 5). The onset of sustained basin formation began around 61.8 Ma, signalling the initiation of compression along the northern Greenland margin that evolved into the ‘Eurekan Stage 1′ deformational event by the Eocene^3, 24^. Geochemical evidence indicates that shortly after this plate restructuring there was a shift in the locus of volcanism^13, 16^ (Fig. 5), with explosive eruptions ceasing at Kap Washington around 61.6 Ma and starting at the Nares Strait around 61 Ma^17^. The age of the lowermost bentonites in the Central Basin (61.596 ± 0.028 Ma), the likely product of one of the final explosive eruptions from the Kap Washington Group, overlap with the age of the Danian-Selandian boundary (Fig. 2). The stage transition has an astronomically calibrated age of 61.607 ± 0.040 Ma^25^. The boundary marks a change to the deformation style within the Eurasian Plate^26^, the termination of 40 million years of carbonate deposition in the North Sea basin, and a shift to siliciclastic deposition related to the uplift and erosion of the Scotland-Shetland area^27^.

In a rigid plate framework, a defined plate boundary between Greenland and Eurasia is traditionally applied at the time of breakup and seafloor spreading, around chron C24N (e.g. refs 28, 29). The time period between C25N and C24N is also a well-documented change in seafloor spreading direction in the Labrador Sea^30, 31^. However, the North Atlantic has experienced a prolonged history of intermittent extension and basin formation events including late Paleozoic-Triassic, Late Jurassic- Early/Mid Cretaceous and Late Cretaceous-Paleocene times^32^. Therefore the implications of rifting and seafloor spreading on the opposite side of Greenland, in the Labrador Sea and Baffin Bay, renders the northern margin of Greenland a unique tectonic setting with implied relative plate motion, or deforming boundaries. Nonetheless, the synchronicity of sustained compression between Greenland and Svalbard with tectonic changes further south along the Greenland-Eurasia margin, including uplift of the Scotland-Shetland area^27^ and widespread shear deformation^33^, indicates a common driving force affecting all margins of Greenland (Fig. 5).

Several triggers for the stress changes at the Danian-Selandian boundary have been proposed, including the first pulse of magmatism from the NAIP^34^ and the propagation and acceleration of seafloor spreading in the Labrador Sea and Baffin Bay^30, 31, 35^. However, the timings of these broadly contemporaneous events are poorly constrained, and the relative contributions of plate motion driving forces remain contentious. The first phase of magmatism from the NAIP appeared in both West and East Greenland around 62 Ma^34^, yet the degree to which this pulse of magmatism is related to changes in plate motions is unresolved. Dating seafloor spreading in the Labrador Sea and Baffin Bay is complicated by breakup volcanism, variable sediment cover, and an unclear transition between continental and oceanic crust. Intraplate rifting in the Labrador Sea probably started in the late Jurassic^36^, yet the first identifiable unequivocal marine magnetic anomalies are C27 in the Labrador Sea^30^ (63.5–62.2 Ma^37^; summary in ref. 38). This led to motion of Greenland around 100 km to the ENE relative to North America^30^ and is therefore likely to be linked to both the deformation on the eastern margin of Greenland and the onset of compression between Greenland and Svalbard.

Most published stage poles for Greenland^30, 31, 39, 40^ inherently predict right-lateral motion between Greenland and Svalbard for the period between C27N-C26N or C27N-C25N (Fig. 5). However, some models^30, 31, 33^ also include a distinct period of Palaeocene transpression to compression between Greenland and Svalbard that fits broadly with our time period. Our chronostratigraphic data suggests that stage rotations should be revisited in the context of restoring all margins of Palaeocene Greenland, including the evidence for the onset and duration of the Central Basin formation. Choices in timescale, in particular the dating of chrons C27, C26, and C25, as well as the rotations for North America and Eurasia, are integral to consistently deciphering the regional kinematics. Our new age date of 61.8–61.6 Ma for the onset of sustained deposition in the Central Basin provides a key radio-isotopic constraint for refining regional plate kinematic models and the onset of compression between Greenland and Svalbard.

## Methods

Bentonite samples were collected from a selection of borehole cores at the core facility of the Store Norske Spitsbergen Grubekompani (SNSG) in Longyearbyen and from *in situ* samples from within the Svea coal mine (Fig. 1). These samples have the advantage of less alteration than surface outcrops and have been logged to <1 m detail by SNSG. The samples are the same as those used in a previous study for the geochemical characterisation of tephra layers^13^, and the reader is referred to that paper for full details of the cores. Each bentonite is labelled with a borehole name (e.g. ‘Am’ for Amelnryggen), followed by a number based on the position of the ash in that core (‘Am1’ is the lowermost layer present). This research focussed on bentonites proximal to core BH9/2005 at Urdkollen (Ur), which has been the focus of several previous studies^8, 10, 41–43^. Sample ‘Bk1’ from Bolterskaret core BH5-2006 is located 7.5 m above the base of the Firkanten Formation, the first formation of the Van Mijenforden Group that overlies the Lower Cretaceous-Palaeocene regional unconformity. Sample ‘Lk1’ from Lunckefjellet core BH14-2012 is 7.1 m above Firkanten base, while sample ‘SvN’ is equivalent to sample ‘Am1’ as the bentonite layer can be traced between both localities within the coal mine, which is 9.2 m above the formation base. Field outcrop evidence show that these three dated samples are the same layer as sample ‘Lj1’ in the Liljevalchfjellet core (BH1-2006) 11.8 m above the unconformity, allowing this core to be used as the basis of a cross-section.

Bentonite samples were mechanically disaggregated and heavy minerals were separated using standard magnetic and heavy liquid techniques at the University of Oslo (UiO) and at the Massachusetts Institute of Technology (MIT). Zircons were selected under an optical microscope, annealed for ca. 72 hours at ca. 900 °C and chemically abraded with HF at ca. 195 °C for 14 hours^44^. The zircons grains chosen for analyses were spiked with a mixed ^202^Pb-^205^Pb-^235^U tracer (Oslo) that has recently been calibrated to the EARTHTIME (ET) 100 Ma solution^45^, to allow direct comparison with ages obtained with the ET2535 tracer solution^46^ (www.earth-time.org) that was used at MIT. After spiking, the zircons were dissolved in HF (+HNO_3_) at ca. 195 °C for 5 days (UiO) and at ca. 210 °C for 48 hours (MIT). Chemical separation^47^ was done for most grains. Details of the mass spectrometric techniques used are presented in detail in previous articles^48, 49^. The raw data were reduced using Tripoli^50^ and analytical errors and corrections (including Th-corrections, assuming Th/U in the magma of 3) were incorporated and propagated using an Excel macro and ET_Redux based on published algorithms^51, 52^. Ages were calculated using ISOPLOT^53^ and with specified decay constants^54^.
